# *Fancb* deficiency impairs hematopoietic stem cell function

**DOI:** 10.1038/srep18127

**Published:** 2015-12-11

**Authors:** Wei Du, Surya Amarachintha, Ozlem Erden, Andrew Wilson, Amom Ruhikanta Meetei, Paul R. Andreassen, Satoshi H. Namekawa, Qishen Pang

**Affiliations:** 1 Division of Experimental Hematology and Cancer Biology, Cincinnati Children’s Hospital Medical Center, Cincinnati, Ohio 45229; 2 Divisions of Radiation Health, College of Pharmacy, UAMS; 3 Department of Pediatrics, University of Cincinnati College of Medicine, Cincinnati, Ohio 45229; 4 Division of Reproductive Sciences, Division of Developmental Biology, Perinatal Institute, Cincinnati Children’s Hospital Medical Center, Cincinnati, Ohio 45229

## Abstract

Fanconi anemia (FA) is a genetic disorder characterized by bone marrow failure, variable congenital malformations and a predisposition to malignancies. *FANCB* (also known as *FAAP95*), is the only X-linked FA gene discovered thus far. In the present study, we investigated hematopoiesis in adult *Fancb* deficient (*Fancb*^*−/y*^) mice and found that *Fancb*^*−/y*^ mice have decreased hematopoietic stem cell (HSC) quiescence accompanied by reduced progenitor activity *in vitro* and reduced repopulating capacity *in vivo*. Like other FA mouse models previously reported, the hematopoietic system of *Fancb*^*−/y*^ mice is hypersensitive to DNA cross-linking agent mitomycin C (MMC), which induces bone marrow failure in *Fancb*^*−/y*^ mice. Furthermore, *Fancb*^*−/y*^ BM exhibits slower recovery kinetics and less tolerance to myelotoxic stress induced by 5-fluorouracil than wild-type littermates. RNA-seq analysis reveals altered expression of genes involved in HSC function and cell cycle regulation in *Fancb*^*−/y*^ HSC and progenitor cells. Thus, this *Fancb*^*−/y*^ mouse model provides a novel approach for studying the critical role of the FA pathway not only in germ cell development but also in the maintenance of HSC function.

Fanconi anemia (FA) is a genetic disorder with at least eighteen FA complementation groups (named A through S) identified thus far^1^,^2^,^3^,^4^. Patients with mutations in any of the 18 genes show a complex FA phenotype manifested by developmental abnormalities, bone marrow (BM) failure and cancer^5^,^6^,^7^,^8^. Among the FA genes, *FANCB*, also known as *FAAP95*, is the only X-linked gene and can therefore be inactivated in females if the X chromosome that contains wild-type *FANCB* is silenced^9^,^10^. It is known that human FANCB is a component of the FA core complex, crucial for FANCL stability and FANCD2 ubiquitination, a key activation step in the FA repair pathway^11^,^12^. The *in vivo* function of FANCB is not known.

One of the most important clinical features of FA is hematological. Children with FA often develop pancytopenia during the first few years of life^5^,^6^,^7^,^8^. Complications of BM failure are the major causes of morbidity and mortality of FA, and approximately 80% of FA patients die from BM failure^13^,^14^. To date, hematopoietic stem cell (HSC) transplantation (HSCT) is considered as the best treatment for BM failure and leukemia in FA patients^5^,^6^,^7^,^15^,^16^. Many studies indicate that FA proteins play specific roles in hematopoiesis by governing the responses of hematopoietic cells to both genotoxic and cytotoxic stresses^13^,^14^. Loss of FA functions causes excessive apoptosis of HSC and progenitor cells (HSC/P) cells leading to BM failure in the early stages of FA^15^,^17^,^18^,^19^,^20^. Specifically, it has been shown that acetaldehyde-mediated DNA damage contributes to the genesis of hematopoietic failure in FA^21^. On the other hand, other studies identified an exacerbated p53/p21 DNA damage response as an important factor in the progressive impairment of hematopoeitsis in FA patients^22^. As the disease progresses, apoptosis as well as genomic instability imposes a selective pressure on FA HSC/P cells. The loss of stem cell fitness in FA HSC/P cells permits the emergence of resistant clones, which can be transformed and thereby lead to leukemia^23^,^24^.

In the present study, we examined the role of FANCB in hematopoiesis using a *Fancb*-deficient mouse model (*Fancb*^*−/y*^) recently generated in our laboratories and demonstrate that inactivation of *Fancb* in mice resulted in a decreased HSC pool and compromised HSC function. Furthermore, we show that the *Fancb*^*−/y*^ mice exhibit slower hematological recovery and less tolerance to myelotoxic stress than wild-type controls. Mechanistically, we observed loss of stem cell quiescence and deregulated expression of genes involved in stem cell function and cell cycle regulation upon *Fancb* gene inactivation.

## Results

### *Fancb* ^*−/y*^ mice exhibit normal steady-state hematopoiesis

We recently generated a *Fancb* mutant mouse model^25^. As *Fancb* is an X-linked gene^9^,^10^, only male mice carrying the mutant allele (*Fancb*^*−/y*^) were used for experiments (Fig. S1). Male *Fancb*^−/y^ mice were infertile and therefore it was not possible to generate *Fancb*^−/−^ females. This infertile phenotype associated with loss-of-function is consistent with other FA mouse models^17^,^18^,^19^,^24^,^26^. However, the genotypes of offspring from crossing *Fancb*^+/−^ females with wild-type males followed predicted Mendelian frequencies, indicating that no embryonic lethality or perinatal lethality was associated with the *Fancb* gene mutation (data not shown).

We next examined the effect of the *Fancb* mutation on steady state hematopoiesis. Analysis of peripheral blood (PB) of 6–8 week-old mice showed a slight increase in white blood cell (WBC) counts in *Fancb*^−/y^ mice than their male WT littermates (Table 1). Red blood cell (RBC) count was normal in *Fancb*^*−/y*^ mice compared to the WT controls. No significant difference was seen in the hemoglobin and hematocrit values between *Fancb*^*−/y*^ and WT mice, although the platelet count was somewhat reduced in the mutant group (Table 1). All other hematological parameters, including total erythrocyte counts, appeared to be normal in *Fancb*^*−/y*^ mice, as compared to the WT littermates. Thus, there is no indication of anemia in these mutant animals under steady state, which is consistent with other FA mouse models^17^,^18^,^19^,^24^,^26^.

### Reduced HSC/P frequencies in *Fancb* ^*−/y*^ mice

**Figure 1:**
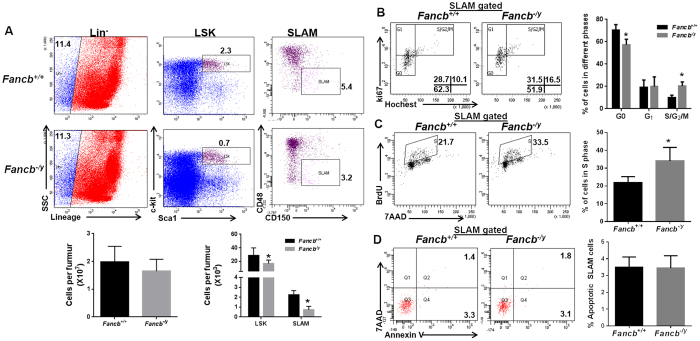
Reduced HSC/P cells in *Fancb*^*−/y*^ mice. (**A**) *Fancb* deficiency reduces the HSC/P pool in *Fancb*^*−/y*^ mice. Whole bone marrow cells (WBMCs) isolated from 8-week-old *Fancb*^*−/y*^ mice or their male Wild-type (WT) littermates were subjected to flow cytometric analysis for LSK (Lin^−^Sca1^+^c-kit^+^) and SLAM (Lin^−^Sca1^+^ c-kit^+^ CD150^+^ CD48^−^) staining. Representative plots (Upper) and quantification (Lower) are shown. Results are means ± standard deviation (SD) of three independent experiments (n = 9 per group). (**B**) *Fancb* mutation increases HSC cycling. Cells described in (**A**) were gated for the SLAM population and analyzed for the cell cycle using Hochest 33342/Ki67 staining. Representative plots (left) and quantification (right) are shown. Results are means ± standard deviation (SD) of three independent experiments (n = 9 per group). (**C**) *Fancb* mutation decreases HSC quiescence. BM cells from WT or *Fancb*^*−/y*^ mice were gated for SLAM population and analyzed for BrdU incorporation. Representative plots (left) and quantification (right) are shown. Results are means ± standard deviation (SD) of three independent experiments (n = 9 per group). (**D**) *Fancb* deficiency does not increase cell apoptosis. Cells described in (**A**) were gated for SLAM population and analyzed for apoptosis by Annexin V and 7AAD. Representative plots (left) and quantification (right) are shown. Results are means ± standard deviation (SD) of three independent experiments (n = 9 per group).

Analysis of different cell compartments in the bone marrow (BM) of the *Fancb*^*−/y*^ mice showed that although the total BM cellularity of *Fancb*^−/y^ mice was comparable to that of WT littermates (Fig. 1A, Lower), *Fancb* deficiency caused a significant reduction in the frequencies of HSC/P cells (Lin^−^Sca1^+^c-kit^+^; LSK) and, importantly, this was also seen in the phenotypic HSC (Lin^−^Sca1^+^ c-kit^+^ CD150^+^ CD48^−^; Signaling lymphocyte activation molecule; SLAM)^27^ compartment (Fig. 1A). Thus, the *Fancb*^−/y^ mice have a reduced HSC/P pool compared to WT controls at the steady state.

### Decreased HSC quiescence in *Fancb* ^*−/y*^ mice

Since quiescence is an important feature of HSC homeostasis^28^, we next analyzed the cell cycle profile of *Fancb*^−/y^ HSCs. Hochest 33342/Ki67 staining showed that there was a significant decrease in the number of quiescent (G_0_) and an increase in the number of cycling (S/G_2_/M) SLAM cells in *Fancb*^−/y^ mice compared to WT control animals (Fig. 1B). We also performed an *in vivo* BrdU incorporation assay to determine the proliferative status of HSCs in the BM. In line with the cell cycle data, the percentage of SLAM cells in S phase was significantly higher (33.98% ± 3.922 in *Fancb*^−/y^ verse 22.65% ± 1.491 in WT, *p* = 0.0356) in *Fancb*^−/y^ mice than WT animals (Fig. 1C). However, mutation of *Fancb* did not affect the viability of SLAM cells at the steady state, as analyzed by Annexin V/7AAD staining (Fig. 1D). These results suggest that the Fancb protein may play a role in HSC homeostasis, probably by maintaining quiescence.

### *Fancb* ^*−/y*^ HSC/P cells show reduced CFU and repopulating ability

**Figure 2:**
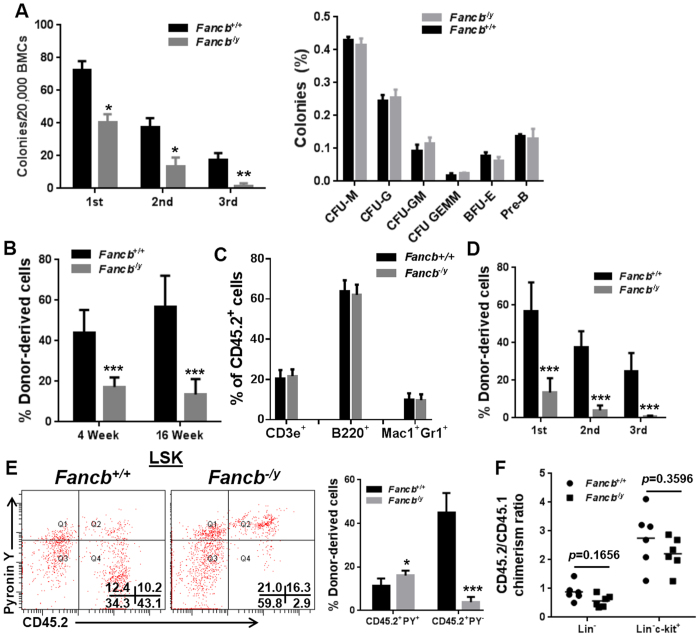
*Fancb*^*−/y*^ HSC/P cells show reduced CFU and repopulating ability. (**A**) *Fancb* deficiency reduces CFU forming capacity. BM cells (BMCs) isolated from *Fancb*^*−/y*^ mice or their male WT littermates were plated in cytokine-supplemented methycellulose medium. Colonies from the 1^st^ plating were pooled for 2^nd^ and 3^rd^ plating. Total colony number (Left) and different types of colonies (Right) were enumerated. Results are means ± standard deviation (SD) of three independent experiments (n = 6–9 per group). (**B**) *Fancb* deficiency impairs the repopulating ability of HSCs. One million WBMCs isolated from *Fancb*^*−/y*^ mice or their male WT littermates (CD45.2), along with equal numbers of congenic WBMCs from BoyJ mice (CD45.1), were transplanted into lethally irradiated BoyJ recipients. Donor-derived chimera was detected by Flow cytometry at 4 week and 16 weeks post BMT. (**C**) *Fancb* deficiency does not alter lineage differentiation *in vivo*. Peripheral blood (PB) from recipients described in (**B**) was subjected to analysis for specific lineages in the donor-derived compartment (CD45.2^+^). (**D**) *Fancb* deficiency impairs the long-term repopulating ability of HSC. Serial BMT was performed by injecting 3 million WBMCs from primary or secondary recipients into lethally irradiated secondary or tertiary recipients, respectively. Results are means ± standard deviation (SD) of three independent experiments (n = 9–10 per group). (**E**) Donor *Fancb*^*−/y*^ HSC/P cells are less quiescent in the transplanted recipients. Cycling donor-derived (CD45.2^+^) cells in the recipients were gated for LSK population and analyzed for cycling status using pyronin Y staining. Representative plots (left) and quantification (right) are shown. (**F**) Effect of *Fancb* deficiency on HSPC homing. Equal numbers of DiO-labelled CD45.2 and DiD-labelled CD45.1 BM cells were transplanted into lethally irradiated CD45.1 recipients (2.5 × 10^6^ from each donor). 16 h post-transplant, the BM of the recipients was analyzed by flow cytometry for the ratio between the percentages of DiO^+^ (donor) and DiD^+^ (competitor) cells within the indicated cell populations.

Increased cycling in *Fancb*^*−/y*^ HSCs may lead to HSC/P exhaustion. To test this notion, we first compared *Fancb*^*−/y*^ and WT progenitor activity using the colony formation unit (CFU) assay. Similar to *Fanca*^−/−^ and *Fancc*^−/−^ mice^29^,^30^,^31^, LSK cells derived from *Fancb*^*−/y*^ mice produced significantly fewer colony formation units than WT LSK cells when plated in methylcellulose supplemented with hematopoietic cytokines (Fig. 2A). However, there appeared to be no bias in lineage differentiation in *Fancb*^*−/y*^ HSC/P cells (Fig. 2A, Right). Significantly, *Fancb*^*−/y*^ LSK cells showed a marked decrease in serial replating activity compared to WT LSK cells (Fig. 2A, Left), indicative of replicative exhaustion in the absence of stromal support.

We next determined the hematopoietic repopulating ability of *Fancb*^*−/y*^ HSCs by performing competitive bone marrow transplantation (BMT). We transplanted whole bone marrow cells (WBMCs) from *Fancb*^*−/y*^ mice or their male WT littermates (CD45.2^+^), along with equal number of WBMCs from congenic BoyJ mice (CD45.1^+^), into lethally irradiated BoyJ recipients. Flow cytometric analysis demonstrated a reduced donor-derived chimera (CD45.2^+^) in the PB of the recipients transplanted with *Fancb*^*−/y*^ cells compared to those transplanted with WT cells (Fig. 2B), which is again consistent with previous reports of impaired repopulating ability of HSCs derived from other FA mouse models^29^,^30^,^31^. However, *Fancb* mutation did not alter lineage differentiation, as we observed similar percentages of donor-derived cells stained positive for CD3ε, B220 and Gr1/Mac1 in the recipients transplanted with either *Fancb*^*−/y*^ or WT cells (Fig. 2C). Furthermore, serial BMT transplantation confirmed a long-term repopulation defect of *Fancb*^*−/y*^ HSCs (Fig. 2D). In corroboration with the cell cycle data from native mice (Fig. 1B,C), we found a significantly decreased proportion of quiescent cells in donor-derived *Fancb*^*−/y*^ LSK cells as compared to WT donor cells (Fig. 2E). Reduced short- and long-term engraftment suggests that *Fancb* deficiency may affect both stem and progenitor cells, perhaps by disrupting a competitive advantage at the early stage of engraftment. To test this notion, we performed a whole-bone-marrow (WBM) homing assay^32^, and found that *Fancb* deficiency decreases homing efficiency (albeit statistically not significant) of stem/progenitor-enriched (both Lin^−^ and Lin^−^c-Kit^+^) cells in the BM of the recipients 16 hours post-transplant (Fig. 2F). Taken together, these results indicate a crucial role of FANCB in maintaining HSC/P function.

### *Fancb* ^*−/y*^ HSC/P cells are hypersensitive to MMC

**Figure 3:**
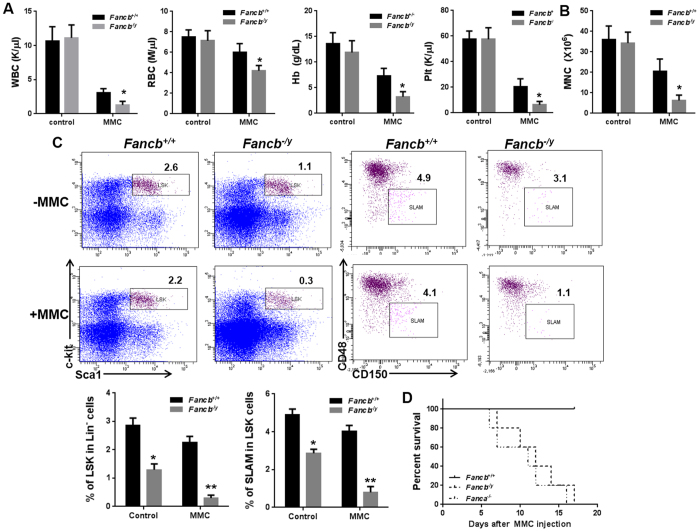
*Fancb*^*−/y*^ HSC/P cells are hypersensitive to MMC. (**A**) *Fancb*^*−/y*^ mice show BM failure after MMC treatment. *Fancb*^*−/y*^ mice and their male WT littermates were injected with 0.3 mg/kg MMC. Hematological parameters were analyzed 8 weeks after MMC injection. Results are means ± standard deviation (SD) of three independent experiments (n = 9–12 per group). (**B**) MMC decreases BM cellularity of *Fancb*^*−/y*^ mice. Total BM mononuclear cells from mice described in (**A**) were isolated and enumerated. (**C**) MMC treatment reduces SLAM and LSK cell number in *Fancb*^*−/y*^ mice. The frequencies of SLAM and LSK cells in WT and *Fancb*^*−/y*^ mice treated with or without MMC were analyzed by flow cytometry. (**D**) MMC treatment leads to the death of *Fancb*^*−/y*^ mice. *Fanca*^−/−^, *Fancb*^*−/y*^ mice and their male WT littermates were injected with a single dose of MMC (1 mg/kg) and survival of the animals was monitored by using the Kaplan-Meier curve method and analyzed by the log-rank test.

BM failure is common in FA patients but does not occur spontaneously in FA mouse models. It has been shown that the DNA cross-linker mitomycin C (MMC) can induce BM failure in *Fancc*^−/−^ mice^18^. We thus examined whether MMC can also induce BM failure in *Fancb*^*−/y*^ mice. Because the hematologic phenotype of *Fancb*^*−/y*^ mice is similar to other FA mouse models and that *FANCA* mutation is most predominant among FA patients^5^, we included the *Fanca*^−/−^ mice for comparison. Eight weeks after chronic exposure to a low dose of MMC (0.3 mg/kg)^18^, *Fancb*^*−/y*^ mice showed severe pancytopenia, as evidenced by significantly decreased RBC, Hb and PLT (Fig. 3A), and BM cellularity (Fig. 3B). In addition, MMC induced marked reduction in LSK and SLAM cells in the BM of *Fancb*^*−/y*^ mice, as compared to WT controls (Fig. 3C). Similar to the *Fanca*^−/−^ mice, all MMC-treated *Fancb*^*−/y*^ mice died within three weeks due to BM failure (Fig. 3D). These results indicate that the hematopoietic system of *Fancb*^*−/y*^ mice is hypersensitive to the DNA cross-linking agent MMC. This is consistent with the shared MMC sensitivity of both mouse and human FA cells to MMC *in vitro*^5^,^6^,^7^,^8^,^18^.

### *Fancb* ^*−/y*^ mice are hypersensitive to 5-FU

**Figure 4:**
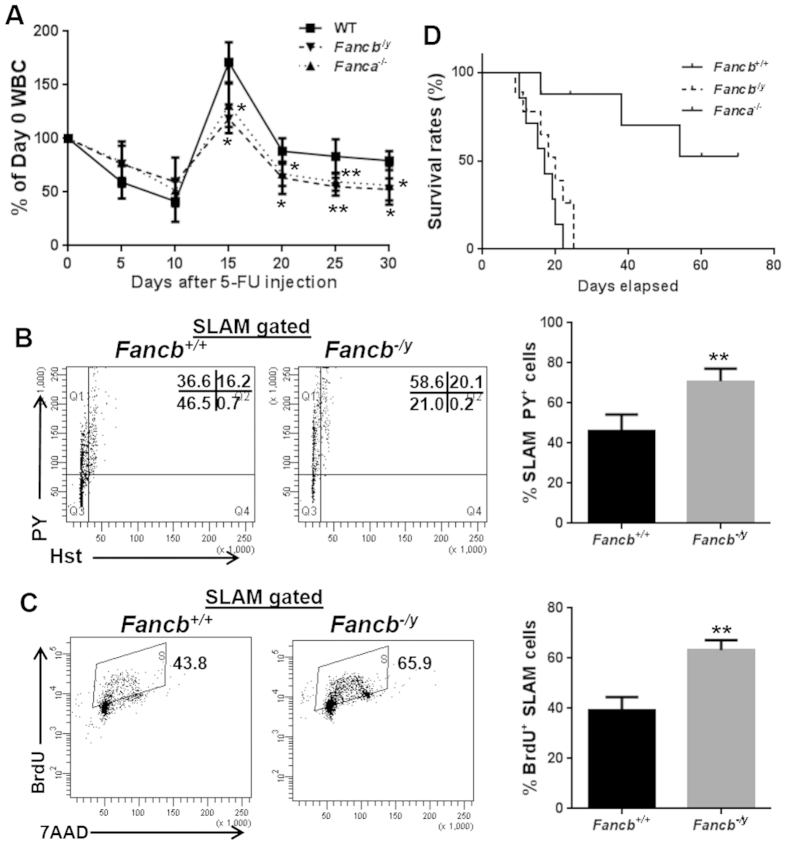
*Fancb*^*−/y*^ HSC/P cells are hypersensitive to 5-FU. (**A**) *Fancb*^*−/y*^ mice exhibit lagging recovery kinetics after 5-FU treatment. The blood cell (WBC) count of *Fanca*^−/−^ or *Fancb*^*−/y*^ mice and their male WT littermates after a single injection of 5-FU (150 mg/kg) was monitored over time. Results are means ± standard deviation (SD) of three independent experiments (n = 6–8 per group). (**B**) 5-FU treatment impairs quiescence of *Fancb*^*−/y*^ HSCs. BM cells were collected from *Fancb*^*−/y*^ mice and WT male littermates 5 days after 5-FU injection, gated for SLAM population and analyzed for the cell cycle using Hochest 33342/Pyronin staining. Representative plots (left) and quantification (right) are shown. Results are means ± standard deviation (SD) of three independent experiments (n = 6 per group). (**C**) 5-FU treatment increases *Fancb*^*−/y*^ HSC cycling. BM cells collected from mice described in (**B**) were subjected to a BrdU incorporation assay. Representative plots (left) and quantification (right) are shown. (**D**) *Fancb*^*−/y*^ mice are hypersensitive to 5-FU treatment. 5-FU (135 mg/kg) was administrated to *Fanca*^−/−^ or *Fancb*^*−/y*^ mice and their WT littermates weekly for 3 consecutive weeks. Survival of the animals was plotted by the Kaplan-Meier curve method and analyzed by the log-rank test.

To further examine the impact of the loss of FANCB on stressed hematopoiesis, we injected *Fancb*^*−/y*^ mice and their male WT littermates, along with the *Fanca*^−/−^ mice as a comparative FA model, with the myeloid-ablating agent Fluorouracil (5-FU) and monitored BM recovery over a period of 30 days^28^. It is known that administration of 5-FU induces hyper-proliferation and exhaustion of HSCs^33^. We found a similar drop of the white blood cell (WBC) count at the first one week after 5-FU injection in both WT and *Fancb*^*−/y*^ mice (Fig. 4A). However, WBC recovery in WT mice started as early as 10 days after 5-FU treatment; whereas the recovery of WBC counts in *Fancb*^*−/y*^ mice persistently lagged as compared to WT mice (Fig. 4A). Of note, *Fanca*^−/−^ mice showed similar lagging recovery pattern (Fig. 4A). Concomitantly, a significantly higher fraction of SLAM cells from *Fancb*^*−/y*^ mice were found in the S/G_2_/M phase of cell cycle (Fig. 4B). Consistently, a significant increase in the number of BrdU^+^ SLAM cell were detected in *Fancb*^*−/y*^ mice over WT mice (63.2% ± 4.03% in *Fancb*^*−/y*^ mice versus 39.225% ± 5.244% in WT mice; *p* = 0.0098) (Fig. 4C). Consequently, 5-FU caused significantly increased mortality of *Fancb*^*−/y*^ and *Fanca*^−/−^ mice compared to WT control animals (Fig. 4D). Thus, like *Fanca*^−/−^ mice, inactivation of *Fancb* renders mice hypersensitive to 5-FU-induced BM ablation, probably due to increased HSC cycling.

### *Fancb* deficiency alters expression of genes involved in stem cell function and cell-cycle regulation

**Figure 5:**
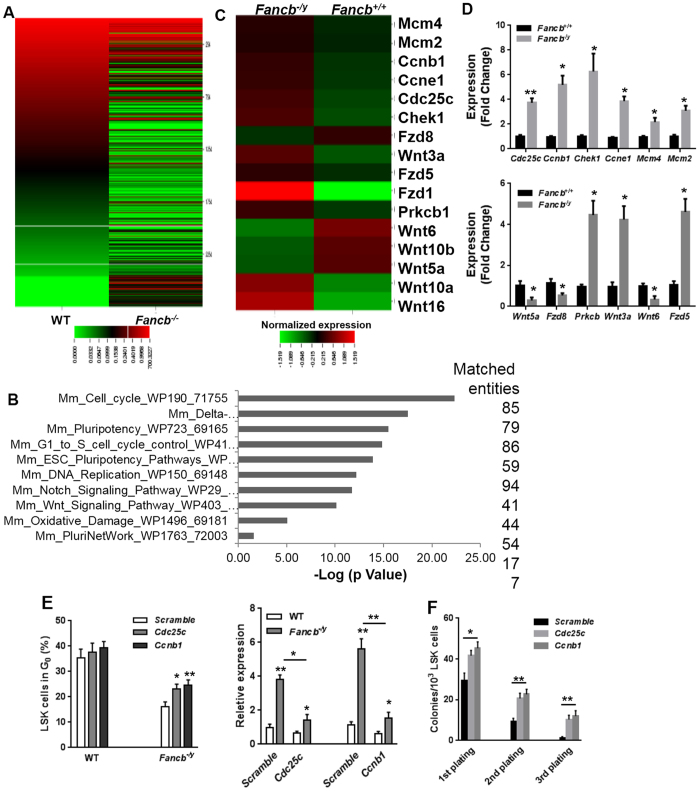
Global transcriptional profiling identifies gene expression changes in *Fancb*^*−/y*^ LSK cells. (**A**) Heat map shows genes that are significantly up-regulated and down-regulated in *Fancb*^*−/y*^ LSK cells. RNA was extracted from LSK cells, isolated from *Fancb*^*−/y*^ mice and their male WT littermates. The mRNAs were analyzed by RNAseq to determine transcripts abundance and differential expression using GeneSpring GX (FC ≥ 2.0, p ≤ 0.05). (**B**) Top 10 deregulated pathways determined by GeneSpring Pathway analysis module using the Wiki Pathway curated database. The X-axis shows -Log10 of *p* value and the Y-axis describes the pathways’ name. The numbers of the matched entities involved in each pathway were shown on the right. (**C**) Heatmap representation of mean normalized expression values of cell cycle regulation and Wnt signaling deregulated genes identify by RNAseq. (**D**) Quantitative RT-PCR validation. RNA extracted from LSK cells, isolated from *Fancb*^*−/y*^ mice and their male WT littermates was subjected to quantitative RT-PCR using primers listed in the Table S1. (**E**) Knock-down of *Cdc25c* and *Ccnb1* in *Fancb*^*−/y*^ LSK cells increases HSC quiescence. WT and *Fancb*^*−/y*^ LSK cells were transduced with scramble, *Cdc25c* or *Ccnb1* lentiviral shRNAs. Twenty-four hours post-transduction, cells were stained with Pyronin Y and Hoechst 33342, and analyzed by FACS. Values are means ( ± SD) (n = 6). The effectiveness of the knockdown for each shRNA is shown on the right. Data are expressed as relative expression relative to the average level in Scramble shRNA-transduced WT cells (set to 1). (**F**) Effects of downregulating *Cdc25c* or *Ccnb1* expression on serial replating of HSPC cells in methycellulose assays. Values shown are means ( ± SD) (n = 3).

To explore the molecular mechanisms underlying the compromised HSC function observed in *Fancb*^*−/y*^ mice, we performed RNA sequencing (RNA-seq) analysis using LSK cells isolated from *Fancb*^*−/y*^ mice and their male WT littermates. Sequencing data were aligned using Tophat and the mm9 version of the mouse genome. Using GeneSpring GX analysis, we found 2753 unique differentially expressed genes (Fig. 5A), of which 973 were up-regulated and 1780 were down-regulated in *Fancb*^*−/y*^ LSK cells as compared to WT cells (Moderate T-test, FC ≥ 2.0).

To further characterize the differentially expressed genes in *Fancb*^*−/y*^ HSPCs, we performed pathway analysis and found that the affected pathways in *Fancb*^*−/y*^ HSPCs included Pluripotency (86 transcripts), cell cycle regulation (85 transcripts), Wnt signaling pathway (54 transcripts), and the Delta-Notch pathway (79 transcripts). Furthermore, the pathway analysis highlighted the role of Fancb in DNA replication (41 transcripts) and oxidative damage (17 transcripts) (Fig. 5B). Further classification based on functional annotation revealed that several differentially expressed genes in *Fancb*^*−/y*^ LSK cells are involved cell cycle control (*Cdc25c, Ccnb1, Chek1, Ccne1, Mcm4, Mcm2*), consistent with the increase in cycling HSCs observed in *Fancb*^*−/y*^ mice. Another group of genes with altered expression was those involved in HSC function (*Wnt10a, Wnt16, Wnt3a, Fzd1, Fzd5, Fzd8, Prkcb*) (Fig. 5C). We validated the RNA-seq results by quantitative RT-PCR (qPCR) for selected genes. Consistent with our phenotypic findings (Figs 1 and 2), many important cell cycle control genes, such as such as *Cdc25c, Ccnb1, Chek1, Ccne1, Mcm4* and *Mcm2*, (Fig. 5D, Upper) and Wnt/pluripotency genes, such as *Wnt5a, Fzd8, Prkcb*, *Wnt3a*, *Wnt6* and *Fzd5*, are deregulated in *Fancb*^*−/y*^ LSK cells (Fig. 5D, Lower). Thus, these transcriptional changes may link dysregulation of multiple pathways to impaired HSC function in *Fancb*^*−/y*^.

Finally, we performed rescue experiments to address whether the transcriptional dysregulation of the cell-cycle related genes in *Fancb*^*−/y*^ LSK cells was associated with the observed increase in HSPC cycling. We chose *Cdc25c* and *Ccnb1*, both of which are critical regulators of cell entry into mitosis^34^,^35^,^36^, for further study. We first acutely lowered the expression of *Cdc25c* and *Ccnb1* in LSK cells, using lentiviral shRNA. We found that reducing the level of either *Cdc25c* or *Ccnb1 *mRNA in *Fancb*^*−/y*^ LSK cells to near WT level (Fig. 5E, Right) significantly increased (albeit not completely rescued) quiescence of the *Fancb*^*−/y*^ LSK cells (Fig. 5E, Left), indicating that dysregulation of these two cell-cycle regulators were at least partially responsible for the decreased quiescence of *Fancb*^*-/*^ HSCs. To examine if knock-down of *Cdc25c* and *Ccnb1* also rescued *Fancb*^*−/y*^ HSC self-renewal, we performed *in vitro* serial replating assays using the shRNA-transduced LSK cells. The downregulation of *Cdc25c* or *Ccnb1* in *Fancb*^*−/y*^ LSK cells significantly increased colony numbers in first plating and, to a greater extent, in second and third platings (Fig. 5F). Thus, genetic correction of *Cdc25c* and *Ccnb1* expression improves *Fancb*^*−/y*^ HSC self-renewal capability.

## Discussion

FA patients suffer BM failure, presumably resulting from depletion of HSCs. Here, we have shown that mice mutated in the *Fancb* gene exhibit defective HSC maintenance. Several observations support this conclusion: 1) *Fancb*^*−/y*^ mice have a reduced HSC pool size at steady state; 2) *Fancb* mutation compromises repopulating capacity of HSCs due, at least partially, to increased HSC cycling and premature stem cell exhaustion; 3) *Fancb*^*−/y*^ HSPCs are hyper-sensitive to genotoxic and myelotoxic stresses; 4) *Fancb* mutation deregulates the expression of genes involved in stem cell function and cell cycle control pathways. This novel mouse model adds to the utility of FA mice for understanding the *in vivo* functions of the FA proteins.

Under steady state, FA mice, including *Fanca*^−/−^, *Fancc*^−/−^ and *Fancg*^−/−^ mice, fail to recapitulate the anemia phenotype of FA patients^17^,^18^,^19^,^24^,^26^. Consistently, our attempt to generate anemic mice by mutational disruption of the *Fancb* gene has not resulted in a model characteristic of the human disease. In fact, *Fancb*^*−/y*^ mice show only minor hematological parameters characteristic of FA patients (Table 1). Although the mechanism behind this is still lacking, possible causes are suggested by the scientific literature. For example, a recent study suggested that mice harbor longer telomere lengths than patients with FA^37^, which might protect hematopoietic cells from senescence. Alternatively, the mild phenotype in mice could be due to fewer spontaneous DNA lesions or lower levels of endogenous DNA-damaging agents, such as formaldehyde^38^ or malondialdehyde^39^.

Although spontaneous BM failure has not been observed in FA mice, a defect in HSC function has been observed in FA mice using competitive repopulation and serial BM transplant studies^19^,^29^,^30^,^31^,^40^,^41^,^42^,^43^. Indeed, we found that loss of Fancb protein compromises HSC repopulation ability and increases proportion of cycling cells in the SLAM compartment, which eventually leads to premature stem cell exhaustion (Fig. 2). Moreover, *Fancb*^*−/y*^ HSCs in lethally-irradiated recipients are less quiescent and are rapidly exhausted upon 5-FU challenge. This correlated with a significantly increased mortality of *Fancb*^*−/y*^ mice (Fig. 4). These results are also consistent with the cell cycle-related HSC defects in *Fancc*^−/−^ mice reported by Li *et al.*^31^. It is in this context that we identify FANCB as a critical regulator that prevents HSCs from myelotoxic stress-induced cycling and excessive proliferation.

It is well-accepted that DNA repair is essential for the maintenance of hematopoietic function. In fact, mice defective in different mechanisms of genome maintenance, including homologous recombination (HR), non-homologous end joining (NHEJ), nucleotide base excision (NER), mismatch repair (MMR), DNA interstrand crosslink repair (ICL), as well as telomere maintenance, have hematopoietic stem/progenitor cell defects^44^,^45^,^46^,^47^,^48^,^49^. Our data on the loss of HSC function in *Fancb*^*−/y*^ mice further supports the importance of genome stability in maintaining the long-term repopulating capacity of HSCs. The impaired regenerative potential of the *Fancb*^*−/y*^ bone marrow may be due to a reduced HSC reserve, functionally defective HSCs, or a combination of the two. In support of a possible role for FANCB in the pathways regulating the balance between stem cell quiescence and cell-cycle entry, our RNA-seq data identified significant difference in gene expression of several particular genes in *Fancb* deficient HSPCs, including those related to cell cycle control (*Cdc25c, Ccnb1, Chek1, Ccne1, Mcm4, Mcm2*) and HSC function (*Wnt10a, Wnt16, Wnt3a, Fzd1, Fzd5, Fzd8, Prkcb*) (Fig. 5). Furthermore, our rescue experiments show that knocking down *Cdc25c* or *Ccnb1 *mRNA in *Fancb*^*−/y*^ LSK cells to near WT level significantly increased quiescence and self-renewal capability of the *Fancb*^*−/y*^ HSCs cells.

In summary, the present study demonstrated that inactivation of *Fancb* in mice induces premature HSC exhaustion and identify excessive HSC cycling as one of the underlying mechanisms for the defect. These findings reveal functional interaction between the FA DNA repair pathway and cell-cycle progression in HSC maintenance. To our knowledge, this is the first study that describes X-linked hematopoietic defects associated with FA.

## Methods

### Mice

Generation of *Fancb* mutant mice has been described elsewhere^25^. Since *Fancb*^*−/y*^ males are infertile, the female was used for mating with C57BL/6J male mice. The offspring were genotyped with the following primer sets: Fancb-WT: 5′-AGAACCATCTGAGGATAAATGT-3′, Fancb-F: 5′-TTTCCAGGTCCCCGTTCTGA-3′ and Fancb-R: 5′-CAAGAATGCGGACTGGAAA-3′ (Fig. S1). *Fanca*^+/+^ and *Fanca*^−/−^ mice were generated by interbreeding the heterozygous *Fanca*^+/−^ mice^26^. 6–8 week-old BoyJ mice were used as recipients for bone marrow transplantation. All the animals, including BoyJ mice, were maintained in the animal barrier facility at the Cincinnati Children’s Hospital Medical Center.

### Treatments

5-FU treatment was adapted from Kobayashi *et al.*, 2004^28^. Briefly, a single dose (150 mg/kg) or multiple doses (135 mg/kg, weekly for 3 weeks) of 5-Fluorouracil (5-FU; Sigma-Aldrich, St. Louis, MO) was administrated intraperitoneally (i.p.) into the experimental mice. Kinetics of BM recovery or the survival of the recipients was monitored.

For *in vivo* mitomycin C (MMC, Sigma-Aldrich, St. Louis, MO) treatment, 6–8 week-old *Fancb*^*−/y*^ mice or their male wild-type littermates were weekly i.p. injected with 0.3 mg/kg of MMC^18^. Blood parameters were measured 8 weeks after the injection. For acute MMC treatment, 1 mg/kg of MMC was used to determine the survival of the subjects^18^. All experimental procedures conducted in this study were approved by the Institutional Animal Care and Use Committee of Cincinnati Children’s Hospital Medical Center according to approved guidelines.

### BM transplantation

1 million whole bone marrow cells (WBMCs) from *Fancb*^*−/y*^ mice or their male WT littermates (CD45.2^+^) along with an equal number of WBMCs from congenic BoyJ mice (CD45.1^+^) were transplanted into lethally irradiated BoyJ recipients. Hematopoietic reconstitution in recipient mice by donor (CD45.2^+^) cells at 8 and 16 weeks post transplantation was determined by staining for CD45.1-PE and CD45.2-FITC markers followed by flow cytometry analysis with a FACSCanto I (BD Biosciences, San Jose, CA). For non-competitive bone marrow transplantation (BMT), 1,000 LSK cells from WT mice or 2,000 LSK cells from *Fancb*^*−/y*^ mice were transplanted to lethally irradiated BoyJ recipient to establish similar chimera. For 2^nd^ BM transplantation, 3 million BM cells from primary recipients were injected to lethally irradiated BoyJ recipients.

### Mouse competitive homing assay

Mouse competitive homing experiments were performed as described^32^. In brief, BM cells from *Fancb*^*−/y*^ or their male WT littermates (CD45.2^+^) were labeled with DiO dye at a density of 2 × 10^6^ cells per ml at 37 °C for 30 min. In parallel, BM cells from BoyJ mice (CD45.1^+^) were labelled with DiD dye (1:200) at 37 °C for 30 min. DiO and DiD were purchased from Vybrant Multicolor Cell-Labelling Kit (Molecular Probes, V-22889). After labelling, dyes were washed off. The DiO-labelled CD45.2 and DiD-labelled CD45.1 cells were mixed at a 1:1 ratio and transplanted into lethally irradiated (11 Gy one day before transplantation) CD45.1 recipients (2.5 × 10^6^ from each donor). Sixteen hours after transplant, the recipients were euthanized and the BM was analyzed by flow cytometry for both DiO/DiD and surface lineage markers (Gr1, Mac1, B220, CD3, Ter119, from Ebioscience) and c-Kit (2B8, BD Biosciences). The ratio between the percentages of DiO^+^ (donor) and DiD^+^ (competitor) cells within different cell populations was quantified.

### Flow cytometry

The lineage marker (Lin) mixture (BD Biosciences, San Jose, CA) for BM cells from treated or untreated mice included the following biotinylated antibodies: CD3ε (145-2C11), CD11b (M1/70), CD45R/B220 (RA3-6B2), mouse erythroid cells Ly-76 (Ter119), Ly6G and Ly-6C (RB6-8C5). Other conjugated antibodies (BD sciences, San Jose, CA) used for surface staining included: CD45.1 (A20), CD45.2 (A104), Sca1 (D7), c-kit (2B8), CD48 (HM48-1), CD150 (9D1). Biotinylated primary antibodies were detected by incubation of antibody coated cells with streptavidin-PerCP or FITC (BD Biosciences, San Jose, CA) in a two-step staining procedure.

For apoptosis staining, cells were stained with Annexin V and 7AAD using the BD ApoAlert Annexin V kit (BD Pharmingen, San Jose, CA) in accordance with the manufacturer’s instruction. Apoptosis was analyzed by quantification of the Annexin V-positive cell population by flow cytometry.

For cell cycle analysis, cells stained for surface markers were fixed and permeabilized with Cytofix/Cytoperm buffer (BD Pharmingen, San Jose, CA) followed by intensive wash using Perm/Wash Buffer (BD Pharmingen, San Jose, CA). Cells were then incubated with Hochest 33342 (Sigma-Aldrich, St. Louis, MO) and anti-mouse Ki67 antibody (BD Pharmingen, San Jose, CA) or Hochest 33342 and 150 ng/ml Pyronin Y (Sigma-Aldrich, St. Louis, MO) followed by flow cytometric analysis.

For the BrdU incorporation assay, Bromodeoxyuridine (BrdU, 150 μl of 10 mg/ml) were intraperitoneally (i.p.) injected to subjected mice followed by BM cells isolation 14 hours later. BrdU incorporated cells (S phase) were analyzed with the APC BrdU Flow Kit (BD Biosciences, San Jose, CA), following the manufacturer’s instructions. Briefly, cells were surface stained then fixed and permeabilized using BD Cytofix/Cytoperm Buffer. After 1 hour incubation with DNase at 37 °C, cells were stained with APC-conjugated anti-BrdU monoclonal antibody. 7-aminoactinomycin (7-AAD) was added to each sample right before Flow Cytometry analysis (BD Biosciences, San Jose, CA).

For cell sorting, lineage negative cells were enriched using lineage depletion columns (StemCell Technologies, Vancouver, BC, Canada) according to the manufacturer’s instructions. The LSK (Lin^−^ c-Kit^+^ Sca-1^+^) population was acquired by using the FACSAria II sorter (BD Biosciences, San Jose, CA).

### Colony-forming unit assay

LDBMCs isolated from *Fancb*^*−/y*^ mice or their male WT littermates were plated in a 35-mm tissue culture dish in 4 mL of semisolid medium containing 3 mL of MethoCult M3134 (Stem Cell Technologies, Vancouver, BC, Canada) and the following growth factors: 100 ng/ml SCF, 10 ng/ml IL-3, 100 ng/ml GM-CSF, and 4 units/mL erythropoietin (Peprotech, Burlington, NC). On day 7 after plating, erythroid and myeloid colonies were enumerated. For serial plating, cells from primary or secondary CFU assays were pooled and re-plated to evaluate secondary or tertiary CFUs, respectively. Hematopoietic clonal growth results were expressed as means (of triplicate plates) ± SD of three experiments.

### RNA sequencing

For RNAseq analysis, RNA was extracted from LSK cells isolated from *Fancb*^*−/y*^ mice or their male WT littermates by Trizol-type method. The RNA quality and quantity assessment, single-end sequencing and alignment of reads on mouse genome (mm9 version) were carried out in the Cincinnati Children’s Hospital Medical Center Affymetrix Core using standard procedures using Illumina HiSeq2000. Bam files provided by the Core were analyzed using Genespring GX v12 (Agilent Technologies, Santa Clara, CA). Quantification of mRNA expression was done using the RefSeq database. Gene expression and fold change (FC) were evaluated between WT and *Fancb*^*−/y*^ samples and genes with a significant difference (moderate-T test, p ≤ 0.05) and a FC ≥ 2.0 were selected to run the pathway analysis module of GeneSpring GX v12 using the curated WikiPathway database(http://www.wikipathways.org).

### Molecular cloning and materials

Hairpin sequence targeting *Cdc25c* (ACCTGAATCTCCGAAAGACAAA) or *Ccnb1* (CCCTCTGTAGTGAATATGTGAA) were cloned into SFLV-eGFP-shRNA vector (Dr. Lenhand Rudolph (Institute of Molecular Medicine and Max-Planck-Research, Germany)^50^. The plasmids (10 μg each) were used to produce retroviral supernatant.

### Statistics

Data were analyzed statistically using a Student’s *t* test. Statistical measures stated in the text were based on the *p* values. *p* values < 0.05 were considered statistically significant.

## Additional Information

**How to cite this article**: Du, W. *et al.* *Fancb* deficiency impairs hematopoietic stem cell function. *Sci. Rep.* **5**, 18127; doi: 10.1038/srep18127 (2015).

## Supplementary Material

Supplementary Information

## Figures and Tables

**Table 1:** Hematopoietic parameters.

	Absolute and differential WBC counts				Characterization of red blood cells				Plts
	WBC (cells/ul)	%Lymphocyte	%Neutrophils	%Monocytes	RBC count (×1012/L)	HCT, %	MCV, fL	Hb (g/dL)	(× 109/L)
*Fancb*^+/+^	6.96 ± 0.76	81.86 ± 4.34	11.74 ± 1.23	2.46 ± 0.70	11.2 ± 1.21	52.6 ± 2.69	55.64 ± 3.86	16.64 ± 1.29	740 ± 89.01
*Fancb*^*−/y*^	7.00 ± 0.96	83.24 ± 5.53	12.84 ± 2.65	3.12 ± 0.68	12.22 ± 1.29	51.72 ± 3.11	50.52 ± 2.6	16.65 ± 1.69	527 ± 94.8
*p*	0.42	0.11	0.47	0.09	0.40	0.20	0.18	0.11	0.04

*p* values were determined using Student *t* test.

WBC count indicates white blood cell count; % Lymphocytes, percentage of WBC count that are lymphocytes; % of Neutrophils, percentage of WBC count that are neutrophils; % of Monocytes, percentage of WBC count that are monocytes; RBC count, Reb blood cell count; HCT, hematocrit (percentage of whole blood volume); MCV, mean cell volume, Hb, hemoglobin concentration; Plt, Platelet count.

For all tests on wild-type mice, the sample size was 10. For all tests on *Fancb*^*−/y*^ mice, the sample size was 9.
