# Supplementary material for: Fancb deficiency impairs hematopoietic stem cell function
Source: Sci Rep. 2015 Dec 11;5:18127. doi: 10.1038/srep18127 (PMC4676042; doi:10.1038/srep18127)
Supplement: Supplementary Information [file srep18127-s1.doc]

***Fancb* deficiency impairs hematopoietic stem cell function**

Wei Du^1,2^, Surya Amarachintha^1^, Ozlem Erden^1^, Andrew Wilson^1^, Amom Ruhikanta Meetei^1,3^, Paul R. Andreassen^1,3^, Satoshi H. Namekawa^3,4^, Qishen Pang^1,3^

^1^Division of Experimental Hematology and Cancer Biology, Cincinnati Children's Hospital Medical Center, Cincinnati, Ohio 45229, ^2^Divisions of Radiation Health, College of Pharmacy, UAMS, ^3^Department of Pediatrics, University of Cincinnati College of Medicine, Cincinnati, Ohio 45229, ^4^Division of Reproductive Sciences, Division of Developmental Biology, Perinatal Institute, Cincinnati Children's Hospital Medical Center, Cincinnati, Ohio 45229

**Address correspondence to:** Qishen Pang, Division of Experimental Hematology and Cancer Biology, Cincinnati Children's Hospital Medical Center, 3333 Burnet Avenue, Cincinnati, Ohio 45229. Phone: (513) 636-1152. Fax: (513) 636-3768. E-mail: [Qishen.pang@cchmc.org](mailto:Qishen.pang@cchmc.org) or Wei Du, Division of Radiation Health, College of Pharmacy, UAMS, 4301 W Markham, Little Rock, Arkansas 72205. Phone: (501)-526-6990. E-mail: wdu@uams.edu

**Supplementary Material**

**
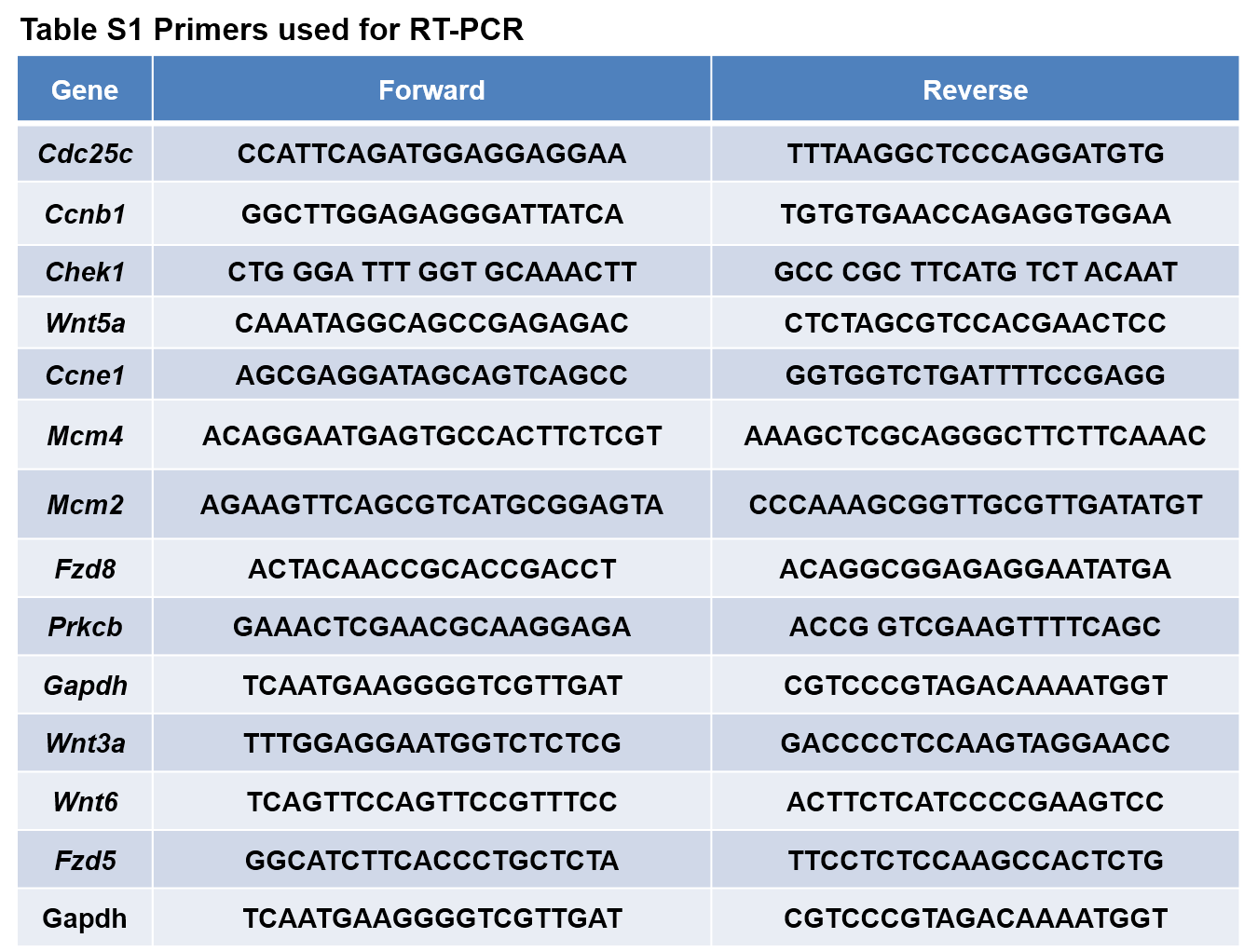
**

**Supplementary Figure**

**
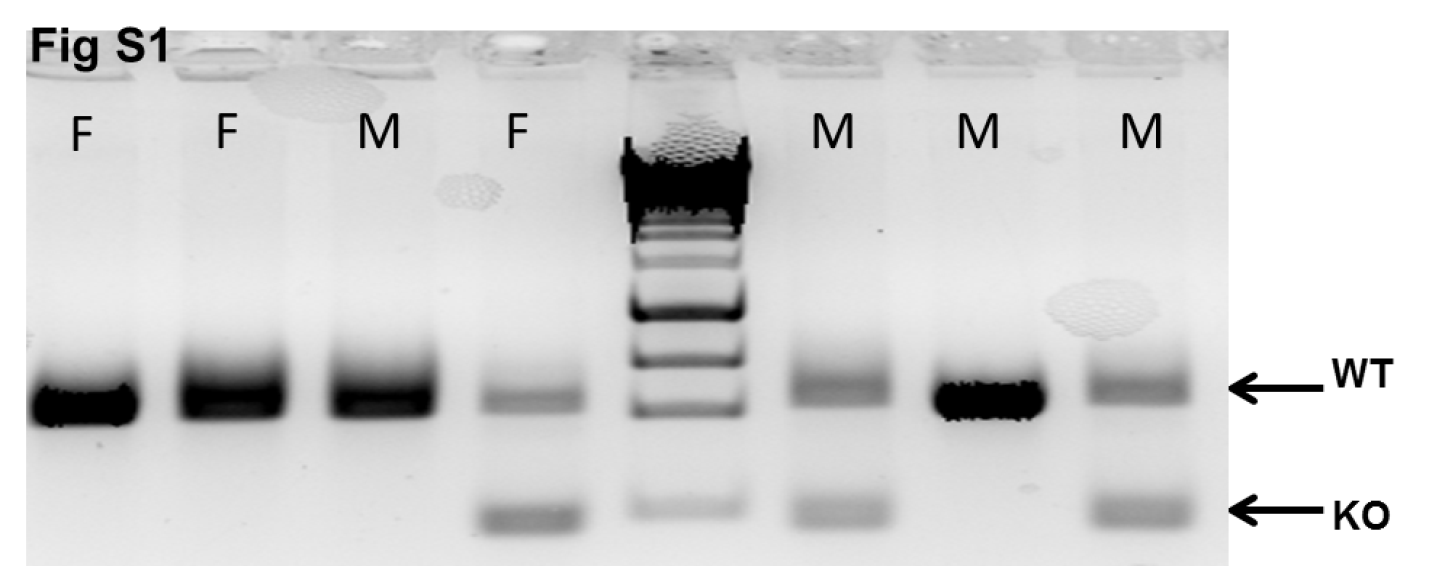
**

**Fig S1.** **Genotyping of *Fancb^-/y^* mice.** Genomic DNA was extracted from tails of the same litter followed by PCR using the primers listed in the Materials and Methods. Male offspring with KO band at 300 base pair (bp) were used for experiments. PCR product at 511 bp was used as PCR control.
